# The cultural attitudes of a funeral ritual discourse in the indigenous Torajan, Indonesia

**DOI:** 10.1016/j.heliyon.2022.e08925

**Published:** 2022-02-09

**Authors:** Anastasia Baan, Markus Deli Girik Allo, Andi Anto Patak

**Affiliations:** a Universitas Kristen Indonesia (UKI) Toraja, South Sulawesi, Indonesia; b Faculty of Languages and Literature, Universitas Negeri Makassar, South Sulawesi, Indonesia

**Keywords:** Cultural attitudes, Toraja ethnic, *Ma'pasa Tedong* discourse, *Rambu Solo’* ritual

## Abstract

*Ma'pasa’ Tedong* is one of the ritual parts of *Rambu Solo',* a funeral ritual series in Toraja culture, Tana Toraja regency, South Sulawesi province, Indonesia. In this ethnicity, the ritual, *Singgi*' is communicated using a high level of *Tominaa* language. Therefore, this study aims to describe and explain the cultural attitudes of the Toraja ethnic recorded in *Ma'pasa’ Tedong* discourse. This is qualitative research with the hermeneutic approach used to interpret and explain the meaning of *Ma'pasa’ Tedong* discourses that reflect personal identity, social attitudes, and Toraja ethnic beliefs. Data were obtained by recording, interviewing two *Tominaa* inhabitants, and conducting a document study. The data were analyzed qualitatively by interpreting the meaning through the following stages (a) understanding the speech, (b) drawing, adjusting, and reflecting the results based on concrete actions by the Toraja ethnic group, and (c) interpreting the reflection results under its existence. The results showed that the Toraja ethnic group has self-awareness, tolerance, tenacity, honesty, discipline, and a tough personality. In addition, these personalities underlie politeness, tolerance, care, social, and cooperation in society. The unique and distinctive attitude of this ethnic group is reflected in their belief in carrying out the *Rambu Solo'* ritual. These findings were cultural documents with significant meaning for anyone interested in understanding the *Toraja* ethnic culture. *Ma' pasa Tedong* event contains relevant messages that aid in the community's development and reflect the Toraja ethnic group's culture. As a cultural heritage, its verses contain various ideas and values (meanings) useful for molding and shaping people's character.

## 1 Introduction

The Toraja ethnic is a group of indigenous people living in Tana Toraja regency located in mountainous areas located in the northern part of South Sulawesi province, Indonesia. This region is regarded as a tourist destination due to its beautiful natural panorama and cultural uniqueness. One of the famous cultures in Tana Toraja regency is *Rambu Solo*’; it is a unique funeral ritual because the procession involves numerous people. This ritual is existent to date regardless of its exorbitant costs. This burden is overcome by cooperation because *Toraja's* people embraced the spirit of togetherness (Hoppenbrouwers et al., 2017).

Compared to Torajan culture, the funeral rituals in Vietnamese culture have similarities regarding social values. Both cultures express a form of grief by bringing offerings when cutting; in Vietnamese, they bring money, flowers, and food, while in Toraja, guests and families bring selected Buffalo and Pigs. In addition, the Vietnamese and Torajan, have a spirit and desire to share (respect for others) by sharing food with the community throughout the ritual. Van (2021), in the actual survey of funeral rites of Vietnamese people, shows that a ceremony of worship, a flower, a tear, or a little heartache of those living preparing for the funeral of their loved one, even more or less it also shows human love between the departing (the dead) and the living. Funerals in Vietnam are multi-day affairs that include multiple intricate rituals. Offerings and a photograph of the deceased are placed on an altar by the family. Family and friends pay their respects and provide incense, white flowers, money, and food during this period.

Compared to *Ngaben* funeral rituals in Bali, Torajan culture in death rituals also spends large amounts of money, even up to billions of rupiahs, for a large party that is categorized as a *Rapasan Sapu Randanan*. This ritual must sacrifice the more than twenty-four chosen Buffalo, some pigs, the cost of the sacrifice, food, and any other related costs. Putri (2018) found that the amount of *Ngaben* costs needed ranges from one hundred million up to two hundred million Indonesian Rupiah. Given the large cost incurred, most of the eight communities have a presumption that *Ngaben* must have more funds (*Ngabehin*). Ngaben becomes a label or stamp for wealthy people with such an understanding. With such a stamp or label, of course, economically underprivileged people will not be able to perform their obligations to their ancestors.

Torajan funeral ritual has a similarity with Tiwah in Kalimantan. According to Schiller (1997), performing the Tiwah ritual is difficult. It necessitates extensive and time-consuming planning, as well as substantial finance. Furthermore, the Tiwah procession might last for weeks up to a month. The bones excavated from the tomb is cleaned by a unique procedure and placed into Sandung as part of this Tiwah ritual. However, the first ceremony spears sacrificial animals like Buffaloes, Cows, and Pigs. The feast of death in Torajan also bears similarities in this Tiwah culture. Torajan's *Rambu Solo*’ culture sacrifices a number of Buffalos and Pigs (without Cows) at a hefty price.

Toraja belongs to the ethnic minority, and most of them are Christian. However, *Aluk to Dolo* is the ancestral belief of the Torajan, which several Torajan community groups still hold. Muslims and Christians are involved in every traditional ritual, and there is a process of inculturation, although the Government recognizes this belief as an aspect of the Hindu Religion. Socially, this ethnic group is associated with certain cultural peculiarities such as wood carving, traditional houses (*Tongkonan*), music or dance, religion, language, and economy. They are well-known for an extremely indigenous ritual tradition, namely *Rambu Solo'*. This ritual is a funeral ceremony originating from the *Aluk to Dolo* belief (Adams and College, 1993). It is regarded as an important social event for the Toraja ethnic group.

According to Syarif et al. (2016), *Rambu Solo'* is a tradition, usually performed during funeral ceremonies. This ritual requires the grieving family to organize a party to signify their last respects to the deceased (Tsintjilonis, 2000b). Etymologically, the term of *Rambu Solo'* is derived from the word of *Rambu,* which means "smoke or light" and *Solo'* meaning "down". Therefore, *Rambu Solo'* is interpreted as a ceremony held when the sunsets. This ritual is also referred to as *Aluk Rampe Matampu'*. *Aluk* means 'belief, ' *Rampe* means 'one side or part', and *Matampu'* means 'west.' Conversely, this ceremony is held in the western part of the traditional house (Zerner, 1981).

Presently, funeral rituals are still observed by the Toraja ethnic group. They have preserved these customs and culture. Besides, their social life is bound by the prevailing customs, which has led to these ritual ceremonies' continued existence. The people still follow the rules that have been applied from generation to generation when performing funeral rituals (Wellenkamp, 1988). These unique and exciting stages are symbolic and contain visual and audiovisual elements such as architecture, art, and language. It is believed that the *Rambu Solo’* ritual ceremony is the last tribute paid to the deceased before sending them off to a more sacred place.

The funeral ceremony in Tana Toraja regency is central to symbolic action, especially in distributing slaughtered meat. Funeral rituals are also a story of status, where pride (honor) is at stake (Volkman, 1985). Similarly, Paranoan (1990) explains that the ritual of a funeral ceremony is carried out based on the dignity of a family. Thus, the number of sacrificial animals slaughtered in a *Rambu Solo’* ceremony is a benchmark of high dignity. Funeral ceremonies held in Tana Toraja regency vary greatly depending on the level or social status. At the highest status, they usually sacrifice hundreds of Buffalos; the Buffalo should be the best choice (Idaman, 2012). The implementation of the funeral ceremony is a sign of love for the deceased. Torajan feels cursed if not court their parents, who died properly under the measure of culture (Paranoan, 1990). While, Wahyuningsih (2018) says that the funeral ceremony in Toraja aims to honor and deliver the spirits of the deceased to the spirit realm, along with their ancestors who housed a place called *Puya* (heaven). If the ceremony of the funeral ceremony is enlivened by sacrifices (cattle, e.g., Buffalo) according to the rules of the funeral ceremony, then the sacrifices and crowds will be the provision to the world of origin called *Puya* (Jumiaty, 2013). A Buffalo that is fat, young, and black as a meaningful offering to God. This ritual hopes that God's administration will remain and always keep the man in continuing to live as one of the *Tallu Lolona* philosophy of Torajan who synergize on earth. The custom of the Toraja people to be grateful and grateful for the favors they receive through the greetings of *Kurre Sumanga'* (thank you). Torajan always gives thanks for the land that becomes a legacy for the family to live daily activities, cultivate crops, and hold routine activities, including funeral ceremonies (Sudarsi et al., 2019).

Torajan, who follows the ritual of ancestors, believes that the dead have not been considered dead but are considered as sick people so that the dead are still served food and drink with trays and cups every time people eat the same as when they were children (Said, 2004). The deceased man was placed in a house called *Tongkonan*. This *Tongkonan* is multi-functional. *Tongkonan* is the center of management of various institutions such as religious institutions, kinship institutions, educational institutions, farm links, forestry institutions, and land links (Sandarupa, 2015). *Tongkonan,* for the Torajan, is not just a large family home or traditional house. *Tongkonan* is a place to maintain the alliance of relatives even with long-dead ancestors (Pasande, 2013). In the heat of mourning, the families who came expressed sorrow in various ways. The expression of grief of the Toraja people can be seen from the bating activity. *Bating* in Toraja means lamentation; it is also a deep strand of grief for someone who is abused by death. A person is wailing weeps so much on the side of the corpse on his back while expressing his sorrow with somber words (Herianah, 2012).

Torajan culture in funeral ceremonies gave birth to work ethic in perseverance, hard work, and frugality for implementing rituals among the Torajan. The culture motivates to increase creativity and competence in various areas of life because of cultural demands (Pasande, 2013). Similarly, family values in the life of the Torajan formed in the attitude of daily behavior can create harmonization of life in family and community life (Patiung, 2017). The funeral ceremony is one of the Toraja ceremonies that usually strengthen the value of affection. As a forum for self-actualization to build social relationships between families and communities (Patiung, 2017). The attachment with the group is very strong in Toraja. Personal interests, in many cases, must succumb to the interests of the group for the sake of peace and harmony (Pasande, 2013). The Torajan get along and work with other people. Willingness to sacrifice (cut buffalo) will give birth to social sensitivities that can be useful to fellow human beings; then, the community participates in helping in its implementation (Patiung, 2017).

The people consider implementing the stages involved in the funeral ritual as a tradition to honor the sacred heritage of their ancestors. They are performed in a symbolic form that is visual and verbal in nature. However, these verbal rituals are conveyed through speech in *Torajan* oral literature, referred to as *Kada-kada Tominaa* or *Tantanan Kada* (Scarduelli, 2005)*. Kada Tominaa* is a poem or series of Torajan literary language, usually recited by *Tominaa,* one of the traditional leaders during the *Rambu Solo'* traditional ceremony. Based on their ancestral belief, it is regarded as a form of prayers and offerings carried out by the leader.

The *Toraja* language used in reciting the *Kada Tominaa* differs from that used for daily communication. However, the community usually adopts the ordinary Toraja language. Meanwhile, during the ritual ceremonies, the *Tominaa* language is used. According to Surya et al. (2017), both languages are different, and the people usually communicate in Toraja language. A high level of this same language is also used to recite the *Kada-kada Tominaa*. This language is only spoken and understood by certain people. Therefore, its delivery does not deviate from the situation or ongoing customary event.

Moreover, a ritual activity needs to be performed before the main event begins, known as *Ma'pasa’ Tedong*. Buffalo is the most important animal in their social life in this ethnicity. The 'buffalo market' is an activity carried out to reinvent what the family initially promised. All the buffalos donated by the family are kept at the *Tongkonan* yard, where they intend to bury the corpse (Volkman, 1984).

The *Ma'pasa’ Tedong* ritual procession begins with assessing the buffalos, where the biggest and best of them all named *Parere or Balean* is selected. *Ma'a* cloth is placed on its back, which complements the Tana Toraja regency's various traditional ceremonies and activities. This is considered sacred because religious and customary leaders commonly use it. Subsequently, the *Balean* and the other Buffalos were paraded three times around the *Bala'kaan* in an open field. *Bala'kaan* is a platform erected for funeral ceremonies, and it serves as a place for distributing slaughtered buffalo meat.

In the course of the *Ma' pasa Tedong* event, the speech is delivered in the *Tominaa* language. It is regarded as a *Toraja* oral literature in the form of *Singgi'*. This literary work shower praises both on the Creator and fellow humans based on their position or social status. *Singgi'* adopts a high-level *Tominaa* language with several figures of speech as a means of expressing certain intentions that need not be spoken directly. The figures of speech describe the social classification of the community and possess high aesthetic values (Idaman, 2012).

The speech delivered during the *Ma' pasa Tedong* events contains relevant messages that aid in the community's development and reflect the *Toraja* ethnic group's culture. As a cultural heritage, its verses contain various ideas and values (meanings) useful for molding and shaping people's character. In the past, it implied spiritual wealth and formed a guide and their relation with God and the surrounding community (Suriamihardja, 2006).

The speech delivered during the *Ma' pasa Tedong* events contains relevant messages that aid in the community's development and reflect the *Toraja* ethnic group's culture. As a cultural heritage, its verses contain various ideas and values (meanings) useful for molding and shaping people's character. In the past, it implied spiritual wealth and formed a guide and their relation with God and the surrounding community (Suriamihardja, 2006; Patiung and Suleman, 2020). After a state of calm then the person who utter*s Singgi’* says what he wants to convey in a high-level version of Toraja language or *Tominaa* language (Pakan, 2020), commonly called *Singgi'* with the aim of flattering, greeting guests, and even attracting the attention of the intended people (Palayukan, 2021). In this activity, Patintingan et al. (2019) state that several levels must be adjusted to pay attention to: First; The process of pronunciation of *Singgi'* depends on the type of implementation of traditional ceremonial activities, namely to people who are high socially, of course, different from people who have medium social strata, especially if the implementation of traditional low social literature ceremonies automatically the pronunciation process is in a low category. Second; The process of pronouncing *singgi'* to greet guests who attend under the social strata. When an official or guest comes from various agencies or government structures. Thus, *Singgi',* which was thrown by the cast of *Ma'parapa'* pays attention to the high speech of Toraja language to the concerned, and if the present is a group of nobles, then the row of meanings of the language is also focused on the noble class as an award. Thus if what is present is the social strata of the class bring of course the actor *singgi'* only describes family relationships and family relationships with them. However, today most people who *Ma'parapa'* no longer take these two things into account, but the most important thing for them is whether or not the language spoken by them is good. Thus violating the customary provisions that apply in the Toraja community.

*Ma'parapa'* is carried out at several traditional ceremonies in Toraja, both *Rambu Solo'* and *Tuka'* rituals (Aswar et al., 2020). Like; In the activity "*Ma'pasa' Tedong*", that is, all Buffalo to be slaughtered in a funeral ceremony are collected in the courtyard of the *Tongkonan* (traditional house) and the Buffaloes are decorated in such a way and then taken to the designated place (Handayani, 2021). Arriving there, all the Buffalo and the deceased family surrounded the tent made of *Ongan* leaves three times (Handayani et al., 2020). They sat down, and then the person who *Ma'parapa'* calmed the atmosphere and ended with the delivery of *Singgi'* addressed to the deceased and all the Buffalo that had been collected (Lisda et al., 2021). How to express *Singgi'* in the activity "*Ma'pasa' Tedong*" is done by telling the history of the arrival of Buffalo to the world (Lusi and Yuwanto, 2020), up to Toraja according to the ancestral beliefs of the Torajan (Matana et al., 2020). Moreover, the function of the Buffalo is under their respective types and rules about when and where the Buffalo was slaughtered (Naomi et al., 2020).

Related to the ritual of *Rambu Solo'* (death feast) in Toraja, researchers previously only elaborated on aspects in general in a *Rambu Solo’* feast without specifically investigating each stage that contains the noble values Previously, Sampe (2020); Rima (2019) researched about the types and levels of *Rambu Solo’* in Toraja. In addition, Panggarra (2014) investigated the number of Buffalo and other livestock sacrificed in the type of *Rambu Solo*'s level. Tumirin and Abdurahim (2015) also saw the meaning of many cattle sacrificed in the ritual. The depiction of Torajan life's struggle to conduct the death feast with the biggest material (Handayani, 2021). In this present study, researchers investigated one of the stages in the *Rambu Solo’* that had never been studied before, namely the ritual procession ma' pasa' tedong. Ritual ma'pasa' tedong is very important to be studied because in general, people with different cultural backgrounds have not understood the cultural meaning and purpose of this ritual. Even the Toraja themselves still didn't fully understand it. Skeptically, they perceived that the *Ma'pasa' Tedong* procession was just a show that sought to show the condition of the family background in terms of wealth. They thought this was just a prestige. Through this study, researchers tried to describe the type and noble meaning contained in the ritual *Ma'pasa' Tedong* as an important stage in death feast in Toraja.

However, due to the rapid development of communication and information technology in this era of globalization, the study of oral literature tends to be meaningful and offers relevant benefits associated with cultural preservation. This positively and negatively affects the people (Girik Allo et al., 2020). Peoples’ characters tend to be shaped or molded along with the development and awareness of other cultures. This leads to a shift or even the extinction of indigenous tradition because the influence of more advanced cultures has eroded it. Therefore, the study of oral literature is essential to recognize and document the original culture passed on by previous generations. Concentrating on personal identity, the coherence and similarity of people across lifetimes are famously troublesome, and contending conceptualizations exist inside the way of thinking and brain research (Noonan, 2019). Individual re-recognizable proof, connecting people between specific moments, is the main step in designating legitimacy and fault and allotting freedoms and honors (de Andrade, 2011). Identity is a need, a fundamental inclination. Heginbotham (2021) claims that a feeling of character is fundamental that people cannot be sound, assuming they cannot figure out how to fulfill it. As indicated by the Creator, de Andrade (2011) states that character is an emotional need, both intellectual (consciousness of oneself and the neighbor as various people) and dynamic (the individual must "take choices" utilizing individual flexibility and will). According to the mental perspective, character resembles the character signature.

Personal identity is the lawful significance for deciding proprietorship and scholarly interest in examining and characterizing the ideas of people and the person. As far as the congruity of people, personal identity is for definite ideas to be fundamental for any defense of remunerations and disciplines, future self-concern, moral obligation, pride, lament, or sentimentality (Woike et al., 2020). We likely could not have a reasonable idea of "individual" without some individual progression. For Nematzadeh and Haddad Narafshan (2020), "man" was a "measurable term" that associates activities and their benefits and elements midway in law, yet additionally in sensations of achievement, lament, acclaim, and fault (Johansson, 2017)., the following people are at the core of institutional frameworks like criminal law, property, credit, and protection, deciding both obligation and qualification (Chorro et al., 2017). Personal identity fills in as a getting sorted out the rule that makes worldly viewpoints conceivable; it is vital for assumptions, arranging, and verbose recollecting.

There is a need to carry out analytical and empirical studies to identify and understand the local cultural values that serve as guidelines (Fairclough, 1992). It is also used as a bulwark based on the influence of global cultural residues. A spiritualist culture is the antithesis of a global culture that tends to be liberal and materialist. Therefore, there is a need to oversee the development and influence of global culture to boost development, to uphold human dignity and integrity (Biria and Mohammadi, 2012).

This study aims to describe the cultural attitudes, social interaction, and beliefs of the *Toraja* ethnic group using the qualitative analysis and hermeneutical interpretation of *Ma'pasa’ Tedong* discourse. This study encompasses oral literature related to the *Ma'pasa’ Tedong* discourse and the *Rambu Solo'* ritual ceremony. There is a need for anyone interested in visiting the Tana Toraja regency to understand this study. Besides, the results obtained serve as relevant documents for those interested in studying the Torajan culture further.

## 2 Method

### 2.1 The design of the study

This study was carried out by adopting a qualitative analysis, hermeneutic interpretation, and an *emic* approach. Data were collected through recordings, interviews, and documentation studies, while the recitation of *Ma'pasa’ Tedong* by a *Tominaa* at the *Rambu Solo'* event was recorded. The instruments are used to investigate *Ma'pasa’ Tedong's* meaning as well as reflect the personal identity, social attitudes, and *Toraja* ethnic beliefs. Furthermore, an unstructured interview was carried out to clarify aspects of the speech that were not understood. The unstructured-interview contains questions related to the use of the term in the ritual *Ma'pasa' Tedong,* namely: the genealogy of the commemorated person to all family members during the ceremony, social strata and the number of Buffalo sacrificed, the personal Identity of *Torajan*, the meaning of sacrificing Buffalo in traditional parties, the meaning of promises to sacrifice some buffalos, *Tongkonan* functions, Politeness in *Toraja*, the meaning of Apologies to all the elders present, the attitude of the *Toraja* ethnic group in helping relatives or other people when they experience a disaster, the presence of the relatives in the feast, the unique beliefs: the dead persons need to be treated like they were still alive before being buried, and the purpose of all people need to take good care of the corpse as long as it has not been buried.

### 2.2 Participants

Researchers have examined two of them related to ethnography studies that use very limited research subjects, even only one to two. This is done because this present study also only uses two research subjects. A previous study using only two subjects was conducted by Smith et al. (2003), who examined expertise in practice; it is an ethnographic study exploring acquisition and use of knowledge in anesthesia. Smith et al. (2003) applied a qualitative approach using non-participant observation and a semi-structured interview with anesthetic staff in two British hospitals. In addition, there is also ethnography research that only uses 1 (one) subject of research conducted by Zembylas (2004) that examined the emotional characteristics of teaching; it is an ethnographic study of one teacher.

In this present study, the data regarding the verbal ritual was obtained from two *Tominaa* proficient in chanting the *Ma'pasa’ Tedong.* They are (1) *BN*, aged 60, a traditionally male figure, and (2) *ER*, aged 23, a male student. The following Table 1 is the pseudonym of the brief identity of subjects, *Tominaa* proficient in chanting the *Ma'pasa’ Tedong*:

**Table 1:** Pseudonym of the subjects’ identity.

Pseudonym	Origin	Profession	Age
Mr. B.N.	*Toraja*	Toraja Culturalist, and speaker of *ma'pasa' Tedong* Hymn	60 years old
Mr. E.R.	*Toraja*	Speaker of *Ma'pasa' Tedong* Hymn	23 years old

*BN* was chosen to be interviewed because he is a traditional figure and speaker of the *Ma'pasa Tedong* discourse who understands the funeral ritual procession very well. *Although still young, ER* is the next generation of speakers of the very talented *Ma' pasa' Tedong* discourse. Not all Torajans have the talent to speak a unique language in the funeral ritual of *Ma'pasa Tedong*, because the language used is the register, in this case, the language of the Toraja nobility. There is no formal school to learn the language, *ER* is taught directly by senior traditional leaders to preserve ancestral culture.

### 2.3 The data collection of the study

The procedures involved in data collection are (a) following the *Ma'pasa’ Tedong* ritual procession while recording the speech delivered by *Tominaa,* (b) conducting interviews in order to clarify and understand the speech, (c) collecting documents related to the *Ma'pasa’ Tedong* ritual, and (d) transcribing the recordings and interviews. Subsequently, data analysis was qualitatively carried out in various stages (a) identifying the speeches in *Ma'pasa’ Tedong* that reflect the *Toraja* ethnic group, (b) classifying these speeches according to the focus of the study, (c) categorizing and unitizing the data according to the characteristics of the speech delivered, (e) an emic approach and hermeneutical interpretation of the data was carried out based on the views of the subject being evaluated.

### 2.4 Ethical consideration

All recorded interviews had been stored in a password-protected computer at Universitas Kristen Indonesia (UKI) Toraja, and only authors have direct access to the data. Written documents were stored and locked in a cabinet located at the Research Ethics of UKI Toraja office. This study was approved by the Institutional Review Board (IRB) of Universitas Kristen Indonesia (UKI) Toraja, South Sulawesi, Indonesia, and the protocols used in the study were approved by the Research Ethics of UKI Toraja. Two research subjects were informed consent forms according to institutional guidelines, and the data analysis was approved by the Institutional Review Board (IRB) protocols of UKI Toraja.

### 2.5 Data analysis

The cultural meanings of the various messages and their functions, reflected in the *Ma'pasa’ Tedong* discourse, were deciphered using Ricoeur's hermeneutic principles, consisting of the semantic, reflection, and existential levels. These were based on two perspectives, namely (a) distance and feasibility and (b) comprehension and commitment (Pellauer and Dauenhauer (2002). This study gives a feasible insight of the object under investigation, which was carried out in several phases (a) transition from listening to the speech or witnessing the event to understanding its meaning, (b) transition from understanding the actor or speaker to deciphering the meaning of the recitation, (c) review independently the text based on its designation, replacement or situation, and (d) reference or interpretation of the identification (Kaelan, 2009). Operationally, the interpretation and explanation of the discourse were realized through (a) careful understanding of the *Ma'pasa’ Tedong*, (b) drawing, adjusting, and reflecting on the results realized from understanding the speech based on the facts and concrete actions of the Toraja ethnic group, and (c) the outcome of the reflection under the existent culture.

### 2.6 Validity and reliability

For validity purposes, an unstructured interview was prepared with full thoroughness, and two *Toraja* literary culture experts validated the results and, at the same time, as a lecturer at one of the universities in South Sulawesi, namely *AKS* and *LMS* (Pseudonym). Validated aspects are clarity and directions, presentation and organization of items, items' suitability, adequateness of the content, attainment of purpose, and objective. To minimize any potential biases during the analysis, the extracts were discussed with different researchers (the first and second authors are from Universitas Kristen Indonesia Toraja and the third author is from Universitas Negeri Makassar) Universitas Negeri Makassar. After the results indicated data saturation, the data were declared reliable or consistent; this was accomplished by analyzing and collecting data simultaneously.

## 3 Results

*Singgi'* uttered in the *Ma'pasa’ Tedong* discourse is all about the genealogies and social strata of their deceased's descendants. The message contained in the speech illustrates the Personal Identity of the *Toraja* ethnic group, which still maintains kinship relations, including the genealogies and social strata of the other members of the community. These messages are cited and extracted as follows:

### 3.1 Extract 1: discovering the origins and the social strata of the deceased

Each time the ritual of *Ma'pasa' Tedong* begins, the hymn speaker of *Ma'pasa’ Tedong* will begin by mentioning the deceased's genealogy. Each family will talk with each other, sit together and get to know each other. The Hymn speaker also conveyed the social strata of the family, as spoken by Mr. B.N., the hymn speaker of *Ma' pasa' Tedong;* the speech has been translated from local language to English as follows:

*“Here in this place, I am charged with the genealogy of this family. No one will miss the lineage of this grandmother, as clearly conveyed the connection in Batusura’ village.”*

According to this speech, a tie was placed at the bottom of the page to reveal the genealogy in front of the figures in *Batusura*. The speaker conveys information regarding the genealogy of the commemorated person to all family members during the ceremony. In line with the interview results, Mr. *B.N.* said that from the genealogy, “In *Lembang* or *Batusura* villages, this information helps all the families present to discover their origins and the social strata of the deceased." Moreover, during cultural activities, mainly when performing ritual ceremonies, it was discovered that the *Toraja* ethnic has a self-awareness attitude. This has been used to sustain customary values. However, certain attitudes such as violating these values seem to occur in reality, specifically during ritual ceremonies.

### 3.2 Extract 2: *Ma'pasa’ Tedong* requirements: high social strata and the number of buffalos sacrificed

When performing a ritual in *Toraja*, especially *Ma'pasa' Tedong,* what needs to be considered is the condition. Not all *Torajan* when one of their families died and then performed the ritual *Ma'pasa' Tedong*. Only those who have a high social stratum can carry it out at the expense of a small number of buffalo cattle. This is conveyed in the lyrical excerpt *Ma' pasa' Tedong* by Mr. E.R. translated from the original language, *Toraja* language:

*“O people, man, man! All who enter and gather in the current rituals are great people, having a big name anyway in this area”.*

This speech discloses that not everyone is allowed to participate in buffalo market activities. “The reason being that this ritual is carried out based on two conditions, namely social strata and the number of buffalos sacrificed. Conversely, supposing a community from a low social stratum, they are not allowed to perform this activity, irrespective of the numerous buffalos they can provide. On the contrary, supposing the deceased's descendants belong to the high social status, and they are unable to provide the number of buffalos agreed upon by the villagers, the person concerned is also incapable of carrying out this activity,” as described by Mr. B.N. through interview. Furthermore, in an atmosphere of grief, where several people are in attendance, the *Toraja* ethnic is instilled with good personalities, such as restraint, self-control and they do not force themselves to face reality. It is believed that even in mourning, they need to be able to hide their feelings. This is evident in the following speech.

### 3.3 Extract 3: the tenacious and diligent personal Identity of *Torajan*

So important is the message conveyed in the ritual *Ma'pasa' Tedong* delivered by hymns speaker, be it information about the deceased's families or moral messages for all people present. Thus, the hymn speaker affirms and directs everyone to listen for a moment and interpret his every speech. This is conveyed in Mr. E.R.’s speech excerpt, which has been translated from the local *Toraja* language, as follows:

*“Sit carefully, stay calm, put your ears on, and focus your hearing; listen to this delivery and information!”*

This speech implies that everyone needs to be carefully seated, bearing in mind the situation. It directs everyone present, particularly the relatives, to be seated and remain calm. Based on the interview, Mr. B.N. said, "This is aimed at making them listen to the messages, understanding and remembering the advice given.” The tenacious and diligent personal Identity of *Torajan* is cited in the *Ma'pasa’ Tedong* discourse. These attitudes are embraced by the people hoping that they are bound to receive remuneration from their relatives one day when they die.

### 3.4 Extract 4: sacrificing buffaloes: a form of gratitude to their parents

The *Torajan* family ties are powerful. Whenever a family member faces a challenge, then the whole family will support—included in this ritual *Ma'pasa' Tedong*. The speaker of hymn *Ma'pasa' Tedong* told all the people present about the deceased grandmother's descendants that all her grandchildren were successful in the region. The success obtained was one of them thanks to the prayers and good wishes of the deceased grandmother. This is conveyed in the speech *Ma' pasa' Tedong* by Mr. E.R. translated from *Toraja* language:

*“Hearing the news of this grief, the golden grandchildren of this grandmother is like waking each other up in the midst of sleep in the land of people. The children born are disturbing each other in their beautiful dreams in the region.”*

This speech revealed that the deceased had children and grandchildren during her lifetime; her descendants overseas were successful. During the delivery of the discourse, the speaker reminds the relatives that this success is inseparable from the deceased's prayers, hard work, efforts, and hopes. Accordingly, they have to repay the deceased by performing the traditional ceremonies when the time comes. All children are expected to sacrifice at least one Buffalo as a form of gratitude to their parents. The message conveyed in the following *Ma'pasa’ Tedong* discourse teaches the *Toraja* ethnic group to be honest and disciplined. They are always encouraged to embrace all that was initially conveyed.

### 3.5 Extract 5: relatives promise: sacrificing buffaloes until the traditional feast of *Rambu solo’* is completed

Each time the feast of *Rambu Solo’* is held, it is preceded by a conversation about how many buffalo cattle will be sacrificed. The whole family was present and acknowledged how much they could sacrifice. The conversation was listened to by traditional leaders and the community, as quoted from Mr. E.R.’s speech translated from the following *Toraja* local language:

*“For all relatives, you must take care of and keep the best Buffalo and the choice as best you can. The buffaloes of choice will be slaughtered as long as the traditional feast of rambu solo’ is completed. The promise to the grandmother about the sacrifice of buffalo cattle has been conveyed to the community and indigenous elders.”*

This speech implies that every family capable of donating a Buffalo, needs to take good care of the animal. This continues till when it is slaughtered during the *Rambu Solo'* ritual. The message conveys information related to promises made by the children and relatives of the deceased and witnessed by the traditional leaders. They promised to sacrifice some buffalos during their parents' funeral as a form of gratitude for their success. Under the promise made, Mr. B.N. assumes that “It is expected of them to prepare and take care of the animal for several days, months, or years till the main ceremony is performed, or the deceased is buried.” In the case of leadership, *Torajan* believes that a traditional leader needs to possess a strong personal identity and stance to be upheld by the community for their words and decisions. This is evident in the following speech.

### 3.6 Extract 6: *Tongkonan* functions: a place for family meetings and solving problems

For *Torajan*, *Tongkonan* is a place that functions not only as a place to live but also as a place to consult and solve problems. When families gather in *Tongkonan* usually begins with the words of traditional leaders who start by greeting everyone present and expressing gratitude for the meeting, as well as spoken by speakers of hymns *Ma'pasa' Tedong* translated from *Toraja* language as follows:

*“First of all, I ask permission to those who are given the title of “the highest wood” in this area, who guard and build this area, which becomes the source of solutions and places to convey all problems. May we all stay healthy to be present in this Tongkonan talk about some things.”*

This speech reveals that a story's stance is upheld, supposing it is about the *Tongkonan*, a meeting place for the family where members feel secure. According to the speaker, the leader of a *Tongkonan* needs to be highly respected. *Tongkonan* functions as a place for family meetings and solving problems. Subsequently, in this discourse, the speaker also believes that all family members need to uphold every decision made during the meeting held in the *Tongkonan*. During social interactions, the *Toraja* ethnic group maintains a polite and caring attitude, expressed by their actions and words, and several people have witnessed this.

### 3.7 Extract 7: a polite and caring attitude towards others during social interactions

The speaker of *Ma'pasa' Tedong* hymn has a low-profile character when delivering his speech. He continued to give input and criticism for his use of language even though it was very polite. Surely this has an implied message to all present; this can be seen in Mr. E.R.'s quotation in the translation of the following *Toraja* language:

*“Teach me manners in saying to all scholars and culturalists, I sound too big and right like a gong.”*

This speech teaches them to speak politely when entertaining their guests. The speaker conveys a statement apologetically and politely greets the guests who attended the mourning court and those standing by the graveside during the ceremony. According to Mr.B.N. through interviews, “Politeness is an attitude adopted by the *Toraja* ethnic group as a form of respect. Apart from the verbal speech, being polite to guests visiting Tana Toraja regency is also manifested by offering them betel.” A description of the polite and caring attitudes of Torajan is also reflected in the following speech. There is a revelation that the *Toraja* ethnic groups are extremely respectful and courteous to anyone, irrespective of their social status.

### 3.8 Extract 8: maintaining good relations with close or distant relatives

Before going up to the *Lakkean*/pulpit to provide information, the speaker of the hymn *Ma'pasa' Tedong* asked permission to everyone present, front-back, left-right. This conveys the values of *Toraja* local wisdom in *Ma'pasa' Tedong* ritual, as quoted from Mr.B.N. which has been translated from *Toraja* local language as follows:

*“In an introduction, I ask permission and excuse the person in front of me, behind me, on my right and left. Please permission, I will get on to the Lakkean / pulpit.”*

This speech is interpreted as "Apologies to all the elders present, including those seated at the right-hand and left-hand sides." Mr. B.N., through the interview, said that “This apology is extended to the family members present at the mourning court, although permission is requested from the traditional elders before the speaker stands up and goes up to the *Lakkean* to convey certain information.” *Lakkean* is a type of hut that is high, where the corpse is stored and celebrated for several days till the day of burial. The Toraja ethnic group always maintains good relations with people, including close or distant relatives.

### 3.9 Extract 9: directives to sustain a family relationship

In the procession *Ma'pasa' Tedong*, speakers of the *Ma'pasa' Tedong* hymn announced to all residents, especially the grieving families, to pay attention to the rules of welcoming the Buffalo, there is always meaning in the speech of the hymn, as quoted in Mr.B.N. speech translated from the following *Toraja* language:

*“Look and listen to the rules of the order of these welcome Buffaloes.”*

This speech is interpreted as "please listen to directives to sustain family relationships," according to Mr. B.N., through the interview. The speaker expects all family members to heed the directives or advice given. This is because they witnessed a series of activities. Therefore, it is hoped that all family members understand the meaning of the *Ma'pasa’ Tedong* or 'buffalo market' activity. Subsequently, all of them tend to realize the importance of *Ma'pasa Tedong*. This ritual helps all family members meet and assist one another to provide several buffaloes, thereby maintaining a sense of kinship. This attitude of the *Toraja* ethnic group is also reflected in their high tolerance in helping relatives or other people when they experience disaster. However, they were always present whenever they heard sad news or funeral ritual, irrespective of the distance.

### 3.10 Extract 10: the presence of the relatives as a sign of their sorrow

In the ritual of *Rambu Solo,’* the *Torajan*'s custom is to be present to share their grief with the family. They come in groups and sit together, sharing their taste. Furthermore, speakers of *Ma'pasa' Tedong* hymns greeted them with a welcome greeting and greeted them as an expression of gratitude for their presence from various places. Here is an excerpt of the hymn *Ma'pasa' Tedong* delivered by Mr. E.R. translated from the *Toraja* language as follows:

*“For those present in this mourning court, the whole family came from various places. I convey that you all will remain blessed by God and remain prosperous.”*

This quote recounts a story relating to relatives in distant regions who had to travel to pay their condolences. The speaker further stated that these relatives' presence in the mourning court was a sign of their sorrow, and their arrival is expected to comfort the grieving family. The tolerance attitude of the *Toraja* ethnic group fosters mutual cooperation, thereby easing the burdens of one another, particularly those from the grieving families.

### 3.11 Extract 11: the attitude of mutual cooperation

So important is every stage in the ritual *Ma'pasa’ Tedong* so that the speakers of the hymn *Ma'pasa’ Tedong* convey it to all present in the procession. Family togetherness in supporting each other will further strengthen family ties after the party is over. As implied in the lyrics of the speech *Ma'pasa’ Tedong* by Mr. E.R. translated from the original language, the following *Toraja* language:

*“Nothing will be missed, step by step in this Ma'pasa' Tedong ritual. Thank you for the offering, for the sacrifice of Buffalo brought by all family members.”*

The speech does not need to be missed and has to be delivered to the bereaved family that is bound to perform the buffalo market activities. The speakers' point out that all ritual events have to be performed. It implies that when a family is grieving, all members and close relatives have to give something to the bereaved, namely one or several buffalos. In this context, not all the Buffalo taken care of by the family were used for the *Ma'pasa’ Tedong* activity; only a few were slaughtered under the stipulated criteria.

### 3.12 Extract 12: *Ma'pasonglo’:* a sign of affection sign and final respect to the deceased

One of the processions after *Ma'pasa' Tedong* is finished is *Ma'pasonglo'*. Through this *Ma'pasa' Tedong* speech, the speaker introduces the procession and then hints at its meaning, as in Mr. E.R.'s speech excerpt translated from the following *Toraja* local language:

*“The time has come, I will go up the stairs again, count and mention every buffalo of choice in this mourning court.”*

This speech is expressed as "It is important that family members walk hand in hand like buffaloes, and leave a trail similar to their footprint," as clarified by Mr. B.N. through the interview. In this context, the speakers stated that all the family members participated in the activity after selecting Buffaloes according to their various sizes by the traditional leaders. Furthermore, the number of buffaloes was from tens to even hundreds. During the procession, relatives held a red cloth that was approximately 100 meters long, referred to as *Kaseda*. It was tied around their heads. The cloth was also tied to a sieve that resembles a traditional house such as *Tongkonan* that is later carried around the village. This procession is called *Ma'pasonglo*, and it is a sign of affection sign and final respect to the deceased. Apart from personal and social attitudes, the *Toraja* ethnic group also has certain unique beliefs that are presumed to be true. They believe that dead persons need to be treated like they were still alive before being buried. All family members are obliged to respect the deceased like they were still alive.

### 3.13 Extract 13: respect the deceased: serving foods and drinks, as well as cleaning its body

The mutual respect for the *Torajan* is profound. It not only applies to those who are still alive, but even those who have died are also still appreciated. The *Torajan* honored the families of those who died because they recognized the good services performed while the person was still alive. Thus, the feast of *Rambu Solo’* became the embodiment of such respect. This is evident in the speech of *Ma'pasa' Tedong* hymn pronounced by Mr. B.N. translated from the following *Toraja* language:

*“Before starting, I ask permission to the indigenous leaders, the indigenous stakeholders as a place to ask about all customary matters in this Pangleon village. There is a place to ask questions like a rooster to the customary experts at the stop. Like the traditional procession of Ma'pasa' Tedong that we carry out today, where the whole family is united to finish well traditionally from their dead parent.”*

This quote implies that the corpses need to be praised by the people around the funeral home. Through the interview, Mr. B.N. stated that “all family members around the funeral home need to take good care of the corpse as long as it has not been buried.” This is similar to taking care of a living being by serving them food and drinks and cleaning their bodies. Furthermore, it is also hoped that all family members respect the deceased like they were still alive, for example, asking for permission before carrying out certain activities.

### 3.14 Extract 14: expression of gratitude for the fertile land

The poem delivered by the speakers of the hymn *Ma'pasa' Tedong* contains the meaning of gratitude to God for the fertile land. The land becomes a place for people to plant plants as food for humans and buffalo livestock. Expressions of gratitude for the land as a place to rest and breed Buffalo so that it can be sacrificed in the ritual *Ma'pasa' Tedong* at that time. The excerpt is clearly in Mr. E.R.'s speech which has been translated from the following *Toraja* language:

*“Then, the buffalo grandmother spoke again to the Lord saying "I have found the wilderness, full of grass that is cooled by dew and rain in the heat of the sun. I've never been short of food. The Lord answered, "There are all the grasses, plants as a source of your food and there is even a waterfall there as a release of your thirst." Then, the more grandchildren the buffalo grandmother in the wilderness. In that place, they formed a group of buffaloes and walked through forests, valleys, and a mountain that is a hundred mountains to its name.”*

This quotation briefly depicts that a village is a place of rest. A fertile community is covered with clouds and excessive food to meet the needs of the descendants. Mr. B.N. explained through an interview that, “*Toraja* is a village blessed by God. It is fertile, covered in clouds, as well as an abundance of cattle, particularly buffalos”. The means of livelihood by numerous people from the *Toraja* ethnic is raising Buffalo. This is because the grass is fertile and has abundant water sources.

### 3.15 Extract 15: animals are the provisions made for the corpse to journey to *puya*/heaven

When the procession of *Ma' pasa' Tedong* begins, all families with Buffalo of choice will follow the procession in one direction resembling a group to one place. The group in one direction displayed the best Buffalo, namely *Balian* Buffalo by name. Of course, for the family to be a pride of their ability to carry out the ritual. The name of the deceased will be increasingly known in the community and at the same time show the respect of the family to the parents of the deceased, as in the quotation of Mr. B.N. speech translated from the local language, *Toraja* language as follows:

*O everyone, look, the long road has been stretched. The procession of Ma'pasa' Tedong has begun. Buffaloes of choice has been prepared in this mourning court. Muscular Buffalo provided for deceased grandmother. A chosen Buffalo is Balian Buffalo; this will make the grandmother who died known by name. The purpose of this Balian buffalo sacrifice will be realized today in this place.*

Briefly, this speech means that "The market is visited to select an uncircumcised male Buffalo or *Tedong Balian*." The speaker aids in conveying certain directives to all family members, which helps them select the size of the cattle according to customary provisions, namely the Balian buffalo, an uncircumcised male. Besides, this type of cattle is quite expensive. *Torajan* believe that the more expensive Buffaloes are sacrificed, the easier it is for the deceased to journey to heaven or *Puya*. They believe that these animals are the provisions made for the corpse to journey to *Puya*. Besides the *Balian*, other sizes are sold at as high as 1 billion per head, namely *Bonga, Saleko, Lotong Boko, Todi,* and *Doti* buffalos. These are believed to possess sacred values when they are sacrificed for this ritual event.

### 3.16 Extract 16: *Balian* buffalo prohibits bad things during the *Rambu solo’*

*Torajan* always shows respect not only to their fellow human beings but also to animals and plants. As shown in the hymn *Ma'pasa' Tedong* where the speaker tries to speak to the Buffalo of choice, the *Balian* Buffalo, as it is paraded in the *Ma'pasa' Tedong* ritual. The speaker described the beginning of the name to the presence of the *Balian* buffalo in the mourning court at that time, as spoken by Mr. B.N. in a quote that has been translated from the *Toraja* language as follows:

*“O you who had just entered the land of the round moon, when you were born into the land of the sun, and until now nothing has been able to replace how precious you are. However, when through the consensus of the people, then you are named Buffalo Balian. Then, you are taken and carried by the family's son and declare that you are the one to be sacrificed in this traditional procession.”*

Briefly, this discourse means that "*O*, *Balian* Buffalo *ee*, the traditional ritual *Rambu Solo’* starts today; hopefully, no obstacles were encountered, thereby making it possible for the family expectations to be achieved,” as explained by Mr. B.N. through interview. It is believed that once the *Balian* (the best Buffalo) is slaughtered, before any other activity, no obstacles are bound to be encountered, and it is presumed that the family accomplishes all it desires. Furthermore, it is believed that the *Balian* buffalo prohibits bad things from occurring during the *Rambu Solo.’*

## 4 Discussion

This section categorizes the above sixteen extracts into three. The first category is personal identity attitude of Toraja ethnic consists of extracts 1, 2, 3, 4, 5, 9, and 12. The second category is social attitudes of Toraja ethnic that covers extracts: 6, 7,8, 10, and 11. The last category is belief attitudes of Toraja ethnic, which consists of extracts 13, 14, 15, and 16.

### 4.1 Personal identity attitude of Toraja ethnic

• Extract 1: Discovering the origins and the social strata of the deceased
• Extract 2: *Ma'pasa’ Tedong* requirements: high social strata and the number of Buffalos sacrificed
• Extract 3: The tenacious and diligent personal Identity of *Torajan*
• Extract 4: Sacrificing buffaloes: A form of gratitude to their parents
• Extract 5: Relatives promise: Sacrificing buffaloes until the traditional feast of *Rambu Solo’* is completed
• Extract 9: Directives to sustain a family relationship
• Extract 12: *Ma'pasonglo*’: A sign of affection sign and final respect to the deceased

Sarajlic (2020) revealed that personal identity could relate to one's qualitative identity as a member of a certain group of individuals, defined by some distinguishing characteristic that distinguishes them from others. Unlike numerical identity, qualitative identity focuses on what connects a person to a certain group rather than what distinguishes them from others. It focuses on characteristics that some persons have in common. The personal identity attitude in question encompasses how *Torajan* behaves and reacts when dealing with their inner mood, other individuals, or the environment. This personal identity is interpreted as a trait that is understood based on an individuals' behavior. The *Toraja* ethnic has always tried to understand their genealogy. This is important because it helps to reconnect with unknown family members. It increases the closeness between family members, leading to more effective communication. However, this is a potential vehicle for fostering cultural behavior among family members. Family is the basis for developing cultural values in society (Bahfiarti, 2015). Therefore, every individual in a cultured society needs to understand their genealogy or family tree.

The delivery of information regarding the genealogy and social strata in the *Ma'pasa’ Tedong* discourse provides useful knowledge for all family members. These pedigrees make it possible for individuals to know their position concerning one another. The current development of information technology has made it impossible for the younger generation to recognize their relatives and distant family members (Ekadiani et al., 2019). Conversely, the family tree is the best way to help them identify their relatives. Besides, understanding the family tree boosts the knowledge of relatives. Instead, the cultivation of cultural communication makes it easier for them to discover and develop closer family relationships (Bahfiarti, 2015).

In ritual tradition, the *Toraja* ethnic group always maintains a self-awareness attitude, which everyone possesses. All cultures and even religions recommend that every individual needs to possess this behavior. In this context, it is interpreted as the self-awareness of one's limitations and an understanding that a person's knowledge is constrained (Pacholik-Żuromska, 2019). In social life, it is the basis of true tolerance. This attitude implies that irrespective of the fact that people can carry out certain activities, although supposing it is not following the prevailing tradition, they are bound to adhere to the usual value. Therefore, teaching or knowing self-awareness is important for living a quiet and peaceful life.

This attitude is interpreted as self-awareness, and it is important because it offers a better understanding of people and aids in the unique development of individuals. Understanding themselves causes these individuals to be empowered in certain areas, strengthens them, and identifies their weaknesses. Self-knowledge causes an individual to become aware of their existence and attributes. Social life strategically makes it possible to understand the differences in various social situations (Freitas et al., 2019).

Moreover, when in an uncertain mood and feelings of distress, the *Toraja* ethnic group is also taught to be self-restrained. This means curbing the emotions caused by the pressure of situations and environmental conditions. Although by behaving in this way, individuals do not try to directly change the situation which is the source of the problem, instead they try to analyze the good side, hope for sympathy and understanding from others, or try to forget everything related to the circumstances that have suppressed their emotions (Folkman, 1984). This self-restraint is personal control over oneself and one environment. This attitude has significant benefits such as comfort and self-satisfaction with the environment during social interactions (Samani et al., 2017). However, based on the ability to refrain from this, individuals tend to overcome the physical and psychological disturbances from the environment. Persons that can control and refrain themselves are not affected by environmental conditions.

*Toraja* ethnic group has a principle that states that everything on the earth's surface is a gift from God to humans. Subsequently, only diligent and tenacious humans are bound to benefit from this gift. Likewise, heaven or the hereafter is only provided by God for diligent people that performed good deeds and worshiped God while living in the world. Therefore, a diligent and tenacious personal identity is one of the characteristics of the *Torajan* ethnicity in dealing with all kinds of matters. This is because these traits are perceived as being beneficial, both in world affairs and the hereafter. Every individual's effort to embrace this behavior is an obligation because it facilitates all desires that need to be realized, both in education and society (Tang et al., 2019).

Honesty and discipline in fulfilling promises are essential to the *Toraja* ethnic group. Besides, honesty causes them to embrace praiseworthy behavior, namely facing reality by speaking the truth. The people also have a disciplined attitude, such as prioritizing punctuality in their daily behavior. Honesty and discipline are attitudes that need to be possessed by every individual (Setiawan et al., 2019). These moral attitudes need to be developed in the educational culture both inside and outside school.

*Toraja* ethnic group believes that leaders need to possess a strong personal identity to carry out their duties to protect the community effectively. A tough personal identity makes individuals stronger, more resilient, and optimistic in facing challenges and reduces the negative effects. Therefore, their relationship with others is always valued. Likewise, leaders with a good social attitude with their community have a significant positive effect on increasing their self-esteem (Harris and Orth, 2019).

### 4.2 Social attitudes of Toraja ethnic

• Extract 6: Tongkonan functions: A place for family meetings and solving problems
• Extract 7: A polite and caring attitude towards others during social interactions
• Extract 8: Maintaining good relations with close or distant relatives
• Extract 10: The presence of the relatives as a sign of their sorrow
• Extract 11: The attitude of mutual cooperation

The *Ma'pasa’ Tedong* discourse during the *Rambu Solo'* rituals makes it easier to understand the attitudes and social behavior of the *Toraja* ethnic groups. This social attitude is developed based on individuals' reciprocal social interactions, influencing each other in social life. Interactions between several elements of individual behavior could improve team effectiveness (Hackel et al., 2019). In these interactions, there are norms or rules upheld and obeyed in order for it to occur effectively. These attributes also regulate the attitude and behavior of individuals during the interaction process with other people.

During the *Rambu Solo'* ritual, the *Toraja* ethnic group maintains a polite and caring attitude, particularly when entertaining guests present in a grieving atmosphere. Politeness and caring attitudes are essential elements of daily social life. Conversely, being polite and caring for a person is appreciated. There are certainly some norms or ethics adopted in an individuals' social life when dealing with other people. In these circumstances, polite and caring attitudes lead to several benefits or positive effects on oneself and others (Zamanzadeh et al., 2015). The *Toraja* ethnic group firmly upholds the effort to sustain kinship relations. Kinship is defined as a relationship between each entity with similar genealogy, either through biological, social, or cultural descent (Schneider, 1984; Fox and Robin, 1983). It is the fundamental principle for classifying individual social groups, roles, categories, and genealogies. The context of family relations presented by the *Toraja* ethnic group refers to ideal relationships (kinship due to marital relations) and abstract ones (based on social strata). An ethics code regulates these kinship relationships creates more substantial obligations between those concerned.

Subsequently, this type of relationship involves a tolerant attitude exhibited by *Torajan* when they hear that a member of their family or distant relative has experienced a disaster. This tolerance attitude is manifested in words, deeds, and behaviors that reflect appreciation and respect for others. It also creates a sense of unity, fosters affection, and strengthens kinship, resulting in peace and harmony. Tolerance attitude during social interactions needs to be developed because diverse behaviors and individual traits are observed (Hjerm et al., 2020).

The attitude of cooperation and working together to help others is the basis for implementing the *Ma'pasa’ Tedong* ritual in a series of *Rambu Solo'* ceremonies. The absence of these attributes causes specific difficulties during the ritual event. This is because it is costly. *Torajan* people like to work together as well as help each other. Every community member directly benefits from cooperation, which reduces the workload, resulting in a firmer relationship between brotherhood and togetherness, including peace and harmony. Accordingly, efforts to foster a sense of mutual cooperation and social interaction to help people experiencing difficulties are a way of exhibiting kindness (Zerner, 1981).

### 4.3 Belief attitudes of *Toraja* ethnic

• Extract 13: Respect the deceased: Serving foods and drinks, as well as cleaning its body
• Extract 14: Expression of gratitude for the fertile land
• Extract 15: Animals are the provisions made for the corpse to journey to *Puya*/heaven
• Extract 16: Balian buffalo prohibits bad things from occurring during the *Rambu Solo’.*

Individuals build social spaces based on their own class-conforming cultural practices, and these places reaffirm class-conforming belief systems (Becker et al., 2017). *Toraja* ethnic beliefs are understood based on the ritual of *Ma'pasa’ Tedong*. The attitudes of beliefs and faith are shown when a person is aware of and justifies the truth. The right and wrong of a belief need not be debated because it relates to an individual's inner attitude. Likewise, *Toraja* ethnic group also believes in the *Rambu Solo'* ritual. The growing belief in the local culture reflects the original *Toraja* religion, namely *Aluk Todolo*. It is spiritualism that arises and develops spontaneously with the Torajan. This religion is still in existence and reflects in the various aspects of life (Veen, 1965).

During the *Rambu Solo'* ritual, the *Toraja* ethnic group believes there is a need to pay homage to the deceased, particularly family members or their ancestors. This activity is based on love and respect for the deceased, and it is believed that the dead have a sustainable life and the ability to influence the luck of those living (Waterson, 1993). This is to ensure the sustainable maintenance of the ancestors' welfare, which positively impacts living. Sometimes, spirits request special help or assistance. Therefore, respecting ancestors' social or non-religious function is to foster kinship attitudes such as devotion and continuity of the family tree. Respect for ancestors is carried out in the communities and remains an important component of various ritual practices despite the level of technological, political, or social complexity.

The people of Toraja in South Sulawesi believe that the Buffalo serves as a vehicle that spirits to *Puya* (heaven, or the hereafter). These animals also have a unique position for the Torajan, and it is cultivated and used as a plow tool. This is also considered a sacred animal and a symbol of social status. The Torajan believes that the Buffalo is not only a symbol of prosperity, rather it also depicts the strength of the spirit's ride to nirvana (Tsintjilonis, 2000a). During the death procession, Torajan believes that the more buffalos are sacrificed, the better the deceased's life in the afterlife.

The current research studied several components of culture, specifically beliefs and social practices. As a result, future research can focus on other cultural characteristics, such as values and norms during burial rituals in *Ma'pasa' Tedong*, one of the funeral ritual series. Furthermore, more researchers are encouraged to employ an ethnographic design that holistically explores the lives of Torajan, particularly solo ritual signs, to conduct more comprehensive research on Toraja culture. The application of critical discourse analysis on discourse data analysis and thematic analysis on interview data is then recommended as a data analysis technique for future studies.

## 5 Conclusion

The *Ma'pasa’ Tedong* in a series of *Rambu Solo'*, a funeral ritual, is a cultural discourse. It is a record of diverse cultures, creates awareness, and aids the people to develop their behavior. The speech conveyed in the discourse contains messages with information, directives or advice, and expressions related to the people's cultural behavior and beliefs. The *Toraja*'s personal identity, social attitudes, and ethnic beliefs are recorded in *Ma'pasa’ Tedong* speech. It was taught to relatives in *Toraja* ethnic, particularly for the younger generation during the *Rambu Solo'* ritual. Accordingly, this study's findings are important, meaningful cultural knowledge and insight related to the *Toraja* ethnic culture. This understanding becomes more meaningful when associated with the development of character education local wisdom for the young Toraja generation from generation to generation. This research's implications in culture and literature examine the rituals of *Rambu Solo’* and *Ma'pasa' Tedong.* In literary work, this research has implications for the study of Toraja oral literature. *Ma'pasa' Tedong* is an interesting *Toraja* culture; it is documented literately to keep this cultural heritage sustainable. Scholars or researchers have not done literary documentation on *Ma'pasa' Tedong* ritual. The current research is limited to research on several components of culture, specifically beliefs and social practices. Therefore, this study recommends that Toraja culturalists and language experts hold continuous in-the-form workshops on Toraja culture/local wisdom so that the younger generation can deeply understand their own culture. Educators need to develop teaching materials based on Toraja culture to increase understanding and love for Toraja culture.

## Declarations

### Author contribution statement

Anastasia Baan and Markus Deli Girik Allo: Conceived and designed the experiments; Performed the experiments; Analyzed and interpreted the data; Contributed reagents, materials, analysis tools or data; Wrote the paper.

Andi Anto Patak: Analyzed and interpreted the data; Contributed reagents, materials, analysis tools or data; Wrote the paper.

### Funding statement

This work was supported by Universitas Kristen Indonesia (UKI) Toraja, South Sulawesi, Indonesia.

### Data availability statement

The data that has been used is confidential.

### Declaration of interests statement

The authors declare no conflict of interest.

### Additional information

No additional information is available for this paper.
